# Analgesic Impact of a Popliteal Plexus Block to Standard Adductor Canal Block in Arthroscopic Anterior Cruciate Ligament Reconstruction: A Randomized Blind Clinical Trial

**DOI:** 10.1155/2021/1723471

**Published:** 2021-12-17

**Authors:** Atef Mahmoud, Maged Boules, Joseph Botros, Mohamed Mostafa, Safaa Ragab, Mohammed Alsaeid

**Affiliations:** Faculty of Medicine, Fayoum University, Fayoum, Egypt

## Abstract

**Background:**

Damage to the anterior cruciate ligament (ACL) is crippling and often requires an arthroscopic outpatient surgery. Nevertheless, many patients experience severe pain during the first day after ACL reconstruction (ACLR). The adductor canal block (ACB) has yielded conflicting results for post-ACLR pain relief. This research investigated the effect of a supplemental popliteal plexus block on postoperative pain outcomes compared to a sole ACB.

**Methods:**

Following a randomized design, 60 cases scheduled for knee arthroscopy with ACLR using an ipsilateral hamstring graft were separated into two categories. Subjects in group A (*n* = 30) received an ACB only, while subjects in group B (*n* = 30) received combined ACB and popliteal plexus block (PPB).

**Results:**

We found significant differences between the two groups. The time of the first analgesic request (TFR) was later for the combined ACB and PPB (median 8 h) compared to the ACB only group (median 0.5 h). Morphine consumption was lower for patients who received combined ACB and PPB (median 12 mg) compared to ACB only (median 30 mg). The number of the requested doses was lower for the combined ACB and PPB group (median 3 doses) compared to the ACB only group (median 7 doses).

**Conclusions:**

The addition of PPB to ACB was associated with improved analgesia and a reduced need for opioid-based sedatives following ACLR with an ipsilateral hamstring graft (https://clinicaltrials.gov/ct2/show/NCT04020133).

## 1. Introduction

One of the most common injuries to the knee is an anterior cruciate ligament (ACL) sprain or tear due to trauma [1, 2]. ACL damage is crippling and often requires repair with an arthroscopic method, which is an outpatient surgery. Nevertheless, patients experience severe postoperative pain on the first day after the ACL reconstruction (ACLR) [3, 4]. Efficient postsurgery pain management is an important part of patient recovery that is also crucial for their satisfaction. Psychological factors are important for predicting outcomes of patients who undergo ACLR. There is also a negative association between function and quality of life evaluation [5, 6]. Efforts are ongoing to minimize postoperative muscle weakness and maximize postoperative analgesia [7, 8]. ACLR interventions affect the complex innervation of the affected anatomical parts, including the femoral nerve (including its infrapatellar and saphenous branches), obturator nerve, and tibial and common peroneal branches of the sciatic nerve. The anterolateral (camera), anteromedial (instrumentation), and superomedial ports (fluid channel) used during arthroscopy are innervated by the common peroneal, infrapatellar, and saphenous nerves, respectively [9]. Additional vertical incisions (anteromedial port) may traverse the overspreading nerves like the infrapatellar nerve [10]. Shearing, stripping, and excising forces are applied during hamstring tendon harvesting, while the posteromedial thigh incision at the donor site is innervated by branches of the tibial nerve. Hence, surgery-related parameters like surgery port location and graft origin are of crucial importance for selecting appropriate nerve blocks for multimodal analgesic regimens. Ignoring such factors in the analgesic plan can cause severe postsurgery pain [11]. The adductor canal block (ACB) is a novel method for postsurgery analgesia after knee operations. It provides good analgesia to the medial and anterior aspect of the knee by blocking sensory branches of the saphenous nerve and the nerve from the vastus medialis to the knee [12]. Concerning postsurgery pain management, its effectiveness is similar to the femoral nerve block (FNB) [13–18]. The major benefit of ACB is maintaining, or minimizing quadriceps strength decline, which accelerates ambulating and recovery following knee operations [18]. However, its use in ACLR has produced contradictory results due to the anatomical reasons mentioned above [4, 11, 19–24]. Adding a tibial nerve block can effectively cover the hamstring tendon graft area, but at the expense of leg weakness, which could increase the risk of falling. There are also logistical challenges and time limitations associated with given several injections while maintaining rapid case turnover for ambulatory ACLR procedures [25, 26].

The popliteal plexus block (PPB) is a novel sensory block to the posterior knee compartment that anesthetizes the sensory tibial genicular postobturator nerve branches with a minimal effect on the ankle musculature. Here, we performed a randomized clinical trial (RCT) that combined PPB with the standard ACB in patients undergoing ACLR. We investigated whether this protocol improved postoperative analgesia without affecting motor function compared to the effects of ACB alone.

## 2. Methods

This study was performed at Fayoum University Hospital and enrolled 60 adult cases scheduled for ACLR surgery following approval of the Scientific and Ethical Committee of El Fayoum University Hospitals with study number (D 182) in December 2018. This study was registered at ClinicalTrials.gov (identifier: NCT04020133), and written informed consent was obtained from all participants. Patients scheduled for ACL reconstruction with American Society of Anesthesiologist physical status I/II/III, aged >18 years, and body mass index (BMI) <40 kg/m^2^ were included in the study. Patients were excluded if they refused to participate, were not cooperative, had a BMI >40 kg/m^2^, or were allergic to local anesthetics. We also excluded patients with anticoagulation or bleeding problems, previous nerve dysfunction, swelling or contamination over the injection area(s), and daily morphine consumption >40 mg. Sixty patients were randomly chosen to receive either ACB (group A, *n* = 30) or ACB with PPB (group B, *n* = 30) using random sequence numbers that were hidden in envelopes that were opened in the operating room. Physicians and nurses who were in charge of treating participants and gathering data were not aware of the allocation process. Patient history investigations, routine examinations, and other necessary tests were performed following the local guidelines, including complete blood count, blood glucose, serum urea and creatinine levels, liver function tests, coagulation profile, and electrocardiogram. Before the operation, the visual analogue scale (VAS) was explained to all patients ranging from 0 (“no pain”) to 100 (“worst imaginable pain”). In addition, they were informed about the nerve block interventions. All patients fasted for 6 h before surgery. Those in the intervention group received 0.03 mg/kg of intravenous (IV) midazolam and intravenous (IV) 1 g of cefotaxime to prevent infection. Routine monitoring was performed for all cases, including pulse oximetry, electrocardiography, and noninvasive blood pressure monitoring. General anesthesia was used instead of spinal anesthesia, as the latter may affect the primary outcome of the time of the first analgesic request (TFR). It was induced with 1-2 mg/kg of propofol, 1-2 *μ*g/kg of fentanyl, and 0.5 mg/kg of atracurium. All patients were mechanically ventilated via an endotracheal tube. Anesthesia was continued using oxygen and isoflurane 1-2%. In necessary, 10 mg of atracurium was used every 30 min. If heart rate or mean arterial pressure was increased by >20%, 0.5 *μ*g/kg of fentanyl boluses was repeated. To address postoperative nausea and vomiting, 4 mg of IV ondansetron was administered. Nerve blocks were applied following the randomization scheme. The skin was disinfected, and the adductor canal was located via ultrasound. The transducer was placed anteromedially, nearly at the mid-thigh level, and a sterilized high-frequency linear probe 5Y 12 MHz was used (Phillips HD11, Amsterdam, the Netherlands). For cases where the femoral artery was not obvious, color Doppler scanning was used. After identifying the femoral artery, the probe was moved distally to track the artery to the adductor hiatus to become the popliteal artery. The block needle (Stimuplex; Braun Medical, Bethlehem, PA) was administered in the plane of a lateral-to-medial orientation, then advanced toward the femoral artery. After observing the needle tip at the anterior aspect of the artery, local anesthetic (1 to 2 mL) was administered. Needle repositioning was considered in cases when local anesthetics failed. [27] Next, we moved distally with the artery in the adductor canal in order to enter the adductor hiatus, where PPB was given above the artery. [28] Both blocks were performed using bupivacaine 0.5% (1 mg/kg) + epinephrine (0.05 mg). Following ACLR, the VAS score, need for opioid analgesia and sedation level were measured every 4 h for 24 h. For cases with VAS > 4, rescue analgesia was performed (as morphine per a titration protocol of 3 mg morphine sulfate IV as a bolus dose). If necessary, the injection was repeated every 5 minutes (15 mg) for 4 h or 45 mg per 24 h. The morphine titration protocol was suspended with oxygen saturation <95%, respiratory rate <10/min, the development of sedation (Ramsay sedation scale >2), acute adverse effects (e.g., allergy, marked itching, unusual vomiting, and hypotension with systolic blood pressure <20% of baseline values), or reaching a sufficient level of analgesia.

The primary outcome was the TFR, and the secondary outcomes were cumulative opioid consumption, interval in between doses within 24 h after surgery, number of patients requiring postoperative analgesia, and analgesia quality (according to the VAS), which were evaluated every 4 h for 24 h. For all patients, adverse effects such as vomiting, pruritus, and excessive sedation were documented. Sedation was assessed every 4 h using Ramsay's score, which ranges from 1 (“awake”) to 5 (“aroused only by shaking”). Oversedation was considered as a sedation score higher than 4 and a respiratory rate <8 breaths in a minute, and these patients were admitted to the intensive care unit for monitoring. Patients were given 0.15 mg/kg ondansetron for vomiting [29]. At 24 h after surgery, we measured participants' satisfaction with analgesia using a four-point Likert scale, ranging from 0 (“poor”) to 3 (“excellent”).

### 2.1. Sample Size Calculation

G power version 3 software was applied to estimate the sample size. The minimal sample size was determined to be 52, with 26 subjects in each group, with a statistical power of 0.80 and alpha level of 0.05. This sample size was sufficient to compare the time for analgesic requests following the intervention between the study groups. The sample size was increased by 15% to account for potential attrition bias.

### 2.2. Statistical Analyses

Descriptive statistics are presented as mean (SD) for normally distributed numeric variables, median (interquartile range (IQR)) for nonnormally distributed numeric variables, or frequencies and percentages for categorical variables. Group comparisons were carried out with independent sample *t*-tests for normally distributed numeric variables and Mann–Whitney *U* tests for nonnormally distributed numeric variables or ordinal variables. Chi-square and Fisher exact tests were used for categorical variables. SPSS statistics software (version 26; IBM Corp., Armonk, NY) was used for the analyses, and *p* < 0.05 was considered statistically significant.

## 3. Results

**Figure 1:**
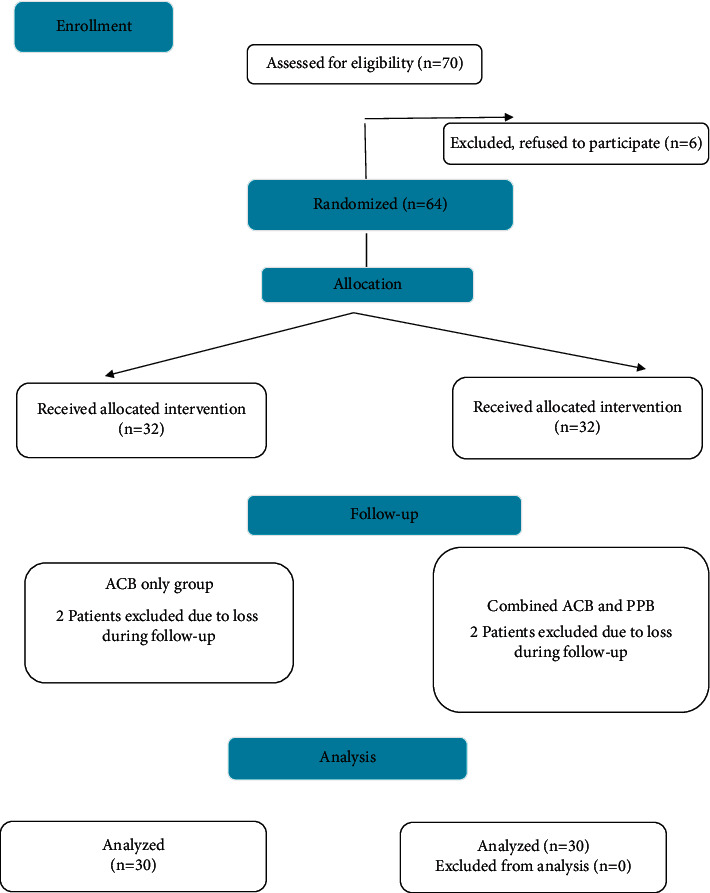
CONSORT flow diagram.

**Figure 2:**
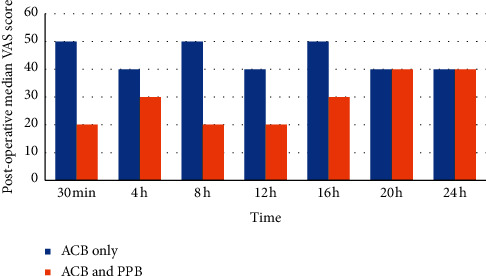
Comparison of postoperative pain scores between groups.

**Figure 3:**
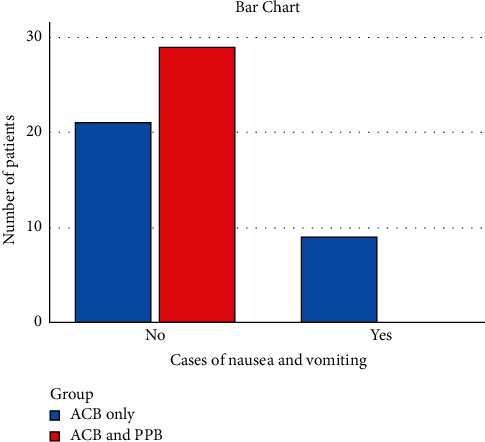
Comparison of postoperative nausea and vomiting.

**Figure 4:**
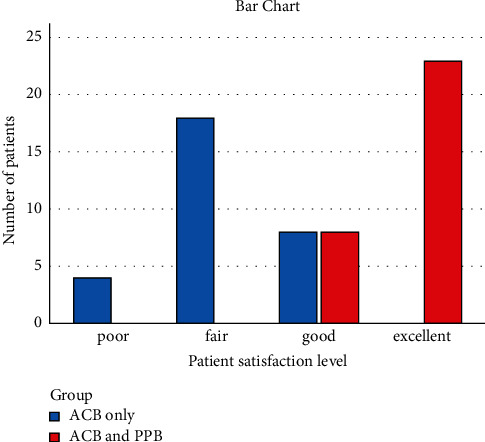
Comparison of postoperative patient satisfaction level.

Seventy patients scheduled for ACLR were admitted to the Orthopedic Department of Fayoum University Hospital between January 2019 and January 2021. Six patients rejected the regional anesthesia method, and the remaining 64 were randomly divided into two groups. Two cases in the ACB only and two in the combined ACB and PPB group were removed due to failed insertion. The study design and final patient cohort are shown in Figure 1. The mean patient age was 24.17 ± 2.28, and the mean BMI was 21.22 ± 2.18 (both *p* > 0.05, Table 1). The TFR, morphine consumption, and number of requested doses were compared. All three variables were significantly different between the study groups (Table 1). TFR was later for the combined ACB and PPB group (median 8 h) compared to the ACB only group (median 0.5 h) (Table 1). Morphine consumption was lower for the combined ACB and PPB group (median 12 mg) compared to the adductor canal block only (median 30 mg) (Table 1). The number of requested doses was significantly lower for the combined block group (median 3 doses) compared to the ACB only group (median 7 doses) (*p* < 0.001) (Table 1). The interval time between analgesic doses was longer in the combined block group compared to the ACB only group (*p* < 0.001) (Table 2). Comparison of the postoperative VAS scores revealed that at each time point from 30 min to 24 h post-ACLR, the median score was lower for the combined block compared to the ACB only (*p* < 0.001) (Table 3, Figure 2). Nausea and vomiting were more common in the ACB only group; only nine cases accounting for 30% of patients had nausea or vomiting compared to none in the combined group, presumably due to higher opioid consumption (Figure 3). Patient satisfaction with analgesia was significantly different between the two groups. All patients in the combined group gave ratings of good or excellent, while no patients in the ACB only group reported excellent satisfaction and only eight cases accounting for 26.7% had good satisfaction (Figure 4). Statistical comparison of the Ramsay sedation scores between groups was not possible, as they did not change (Table 4). No cases of pruritus were observed in either group. As regard, ASA classification in both groups was ASA I in both groups. In regard to gender, in the ACB group, there were was 25 male patients (83%) and 26 male patients in the PPB group (86.6%) with *p* value = 0.730, which was nonsignificant. Surgical time, anesthesia time, and tourniquet time ranged between one and half hours to two hours which was nonsignificant.

## 4. Discussion

The impacted region confirmed in cadaveric studies showed that dye injected in the distal area of the adductor canal stained the tibial genicular nerves, postobturator nerve, and popliteal vessels [28, 30, 31]. Gautier and colleagues used the same technique to inject 20 mL of a solution containing 18 mL of 1% mepivacaine and 2 mL of radio-opaque contrast medium into healthy volunteers prior to computed tomography scans. They reported staining of the popliteal vessels and branches of the sciatic nerves with a minimal effect on the ankle muscles [31].

In their feasibility study, Runge and colleagues used the same approach to evaluate the analgesic impact of adding a PPB to a femoral triangle block (FTB) [32]. They performed unilateral total knee replacement (TKA) in 17 patients with spinal anesthesia using an FTB and evaluated cutaneous sensation and postsurgery pain. The ratio of cases with a numeric rating scale (NRS) score >3 (followed by a decline to <3 after PPB) was defined as the primary outcome. PPB was also administered for 10 (out of 17) cases with a median NRS score of 5.5 (IQR 4–8) following unilateral TKA. For all cases, the NRS was declined to <3 (NRS 1.5 (IQR 0–3)) within a mean time of 8.5 (95% confidence interval 6.8–10.2) minutes. Interestingly, three cases had no pain after receiving PPB. They concluded that PPB was effective in controlling posterior and deep genicular pain after TKA, but the validity of their results was limited by lack of a control group, blinding, and randomization.

Our results are consistent with those of Thobhani and colleagues who used a different approach of local anesthetic infiltration between the popliteal artery and knee capsule (iPACK block) in patients undergoing TKA. They compared a femoral and adductor canal block versus femoral nerve block only. The combined adductor and iPACK block were better than femoral only in terms of adequate control of postoperative pain, especially posterior and deep knee pain. This combination was better than femoral block only in terms of postoperative muscle weakness and length of hospital stay [33]. Similar results were reported by Sankineani and colleagues who concluded that ACB + iPACK is a novel method for improving postoperative analgesia without influencing knee joint motor function, which translated into improved movement compared to ACB alone [34].

The limited efficacy of ACB only in our study was consistent with the results of a meta-analysis by Sehmbi and colleagues [11]. A comparison of ACB to placebo (2 RCTs, 110 cases) revealed that ACB did not increase analgesia following ACLR [4, 19–35]. Nevertheless, a comparison of FNB and ACB (3 RCTs, 308 patients) suggested a potential impact of FNB [13, 36, 37]. It is worth noting that this finding was not confirmed by Mall and Wright [38]. Overall, the low analgesic impact of nerve blocks in ACLR can be attributed to inefficient pain management at graft sites and/or a lack of additional pain relief with multimodal analgesia (MMA) [11]. Ramlogan and colleagues support the routine use of MMA in combination with local infiltration of anesthesia for postoperative analgesia in ACLR and reserve ACB only for patients with opioid contraindications and considering the type of the graft used [22].

Performing combined ACB and PPB with our technique has the advantage that both blocks can be given without changing patient position, saving time in an outpatient setting. It is worth noting the area of pain in the ACB only group moved from a predominantly posterior area to a predominantly anterior or posterior area. However, pain in the combined ACB and PPB group was mainly anterior, reflecting the efficacy of the PPB. Unfortunately, the study was not sufficiently powered to detect changes in the pain site. The second limitation is that we could not evaluate the success of the block as it was done after general anesthesia, but the distribution of the injection was easily seen under ultrasound, which helped us to reduce the incidence of side effects and intravascular injections. The third limitation was short follow-up time. Finally, we did not assess postoperative motor function, as the policy in our institute is to splint patients for 24 h after ACLR. Ultimately, our results are restricted to the study group, procedures, and clinical environments evaluated and cannot be extended to other knee operations, local anesthetic amounts, or analgesic methods.

## 5. Conclusions

The addition of PPB to ACB significantly decreases pain and the need for opioid-based drugs following ACLR with an ipsilateral hamstring graft.

## Figures and Tables

**Table 1:** Comparison of age, BMI, gender, ASA status, morphine consumption, and TFR between groups.

Variable		ACB only	ACB and PPB	*p* value
Age^a^	Mean (SD)	24.1 (2)	24.2 (2.8)	0.823
BMI^a^	Mean (SD)	21.2 (2.3)	21.2 (2.1)	0.953
Morphine consumption (mg)^b^	Median (IQR)	**30.0 (14)**	**12 (4)**	**<0.001**
Number of requested doses^b^	Median (IQR)	**7.0 (3)**	**3.0 (1)**	**<0.001**
TFR^b^	Median (IQR)	**0.5 (3.5)**	**8 (12)**	**<0.001**

^a^Comparison with independent sample *t*-tests. ^b^Comparison with Mann–Whitney *U* tests.

**Table 2:** Comparison of opioid dose intervals between groups^a^.

	ACB only		ACB and PPB		*p* value
	*n*	Median (IQR)	*n*	Median (IQR)	
1st dose interval in hours	**30**	**4 (4)**	**26**	**8 (0)**	**<0.001**
2nd dose interval in hours	**30**	**4 (4)**	**20**	**8 (0)**	**<0.001**
3rd dose interval in hours	30	4 (0)	6	4 (0)	>0.999
4th dose interval in hours	22	4 (0)	0		
5th dose interval in hours	22	4 (0)	0		
6th dose interval in hours	22	4 (0)	0		

^a^Comparison with Mann–Whitney *U* tests.

**Table 3:** Comparison of the postoperative pain scores between groups^a^.

	VAS score	Postoperative pain score 30 min	Postoperative pain score 4 h	Postoperative pain score 8 h	Post operative pain score 12 h	Postoperative pain score 16 h	Postoperative pain score 20 h	Postoperative pain score 24 h
ACB only group	Postoperative pain score 30 min							
	Postoperative pain score 4 h	0.383						
	Postoperative pain score 8 h	>0.999	>0.999					
	Postoperative pain score 12 h	0.233	>0.999	>0.999				
	Postoperative pain score 16 h	>0.999	>0.999	>0.999	>0.999			
	Postoperative pain score 20 h	0.012	>0.999	0.126	>0.999	>0.999		
	Postoperative pain score 24 h	0.012	>0.999	0.126	>0.999	>0.999	>0.999	

ACB and PPB	Postoperative pain score 30 min							
	Postoperative pain score 4 h	0.021						
	Postoperative pain score 8 h	>0.999	>0.999					
	Postoperative pain score 12 h	0.126	>0.999	>0.999				
	Postoperative pain score 16 h	0.029	>0.999	>0.999	>0.999			
	Postoperative pain score 20 h	<0.001	>0.999	0.137	>0.999	>0.999		
	Postoperative pain score 24 h	0.001	>0.999	0.300	>0.999	>0.999	>0.999	

^a^Friedman's test was used as a repeated measures test to study if there is a change in the VAS score at different time points.

**Table 4:** Comparison of Ramsay sedation scores between groups^a^.

Ramsey sedation score (h)	Status		ACB only	ACB and PPB
4	Awake	*n*	30	29
		%	0.508	0.492

8	Awake	*n*	30	29
		%	0.508	0.492

12	Awake	*n*	30	29
		%	0.508	0.492

16	Awake	*n*	30	29
		%	0.508	0.492

20	Awake	*n*	30	29
		%	0.508	0.492

24	Awake	*n*	30	29
		%	0.508	0.492

^a^Statistical comparison of Ramsay sedation scores between the two groups was not possible as they did not change over time.

## Data Availability

The data used to support the findings of this study are available from the corresponding author upon request.

## Abbreviations

abrACB: Adductor canal block
ACL: Anterior cruciate ligament
ACLR: Anterior cruciate ligament reconstruction
FNB: Femoral nerve block
FTB: Femoral triangle block
iPACK: In between popliteal artery and knee capsule block
MMA: Multimodal analgesia
PPB: Popliteal plexus block
TFR: Time of first analgesic requirement
TKA: Total knee arthroscopy.
